# Evaluation of anti-infective-related *Clostridium difficile*-associated colitis using the Japanese Adverse Drug Event Report database

**DOI:** 10.7150/ijms.43789

**Published:** 2020-03-26

**Authors:** Satoshi Nakao, Shiori Hasegawa, Kazuyo Shimada, Ririka Mukai, Mizuki Tanaka, Kiyoka Matsumoto, Hiroaki Uranishi, Mayuko Masuta, Hiroaki Ikesue, Tohru Hashida, Kazuhiro Iguchi, Mitsuhiro Nakamura

**Affiliations:** 1 Laboratory of Drug Informatics, Gifu Pharmaceutical University; Gifu, Japan.; 2 Department of pharmacy, Kobe City Medical Center General Hospital; Kobe, Japan.; 3 Laboratory of Community Pharmacy, Gifu Pharmaceutical University; Gifu, Japan.; 4 Current address: Division of Pharmacy, Nara Medical University Hospital, 840, Shijocho, Kashihara-shi, Nara, 634-8522, Japan.; 5 Current address: Division of Pharmacy, Kyoto City Hospital, 1-2, Mibu Higashitakadacho, Nakagyo-ku Kyoto-shi, Kyoto, 604-8845, Japan.

**Keywords:** *Clostridium difficile*-associated colitis, anti-infective, adverse event, JADER

## Abstract

*Clostridium difficile*-associated colitis (CDAC) may cause gastrointestinal illness, ranging in severity from mild diarrhea to fulminant colitis and even mortality. The purpose of this study was to evaluate anti-infective-related CDAC profiles using the Japanese Adverse Drug Event Report (JADER) database.

**Methods**: We selected case reports of adverse events of CDAC as specified in the Medical Dictionary for Regulatory Activities. The association between the number of administered anti-infectives and aging was evaluated using reporting odds ratio (ROR) and adjusted for covariates using multiple-logistic regression. We also evaluated anti-infective-related CDAC-onset profiles using Weibull shape parameter.

**Results**: The JADER database contained 534 688 reports from April 2004 to June 2018. There were 1222 anti-infective related CDAC events. The top five anti-infectives were as follows: third-generation cephalosporins (Anatomical Therapeutic Chemical (ATC) code: J01DD, 313 cases), fluoroquinolones (ATC code: J01MA, 201 cases), macrolides (ATC code: J01FA, 146 cases), carbapenems (ATC code: J01DH, 143 cases), and penicillins with extended spectrum (ATC code: J01CA, 103 cases). The adjusted RORs (95% confidence interval) in individuals using 1, 2, and ≥ 3 anti-infectives were 8.88 (7.05-11.18), 9.77 (6.89-13.86), and 18.39 (11.85-28.54), respectively. Moreover, 47.2% of CDACs occurred within 7 days of anti-infective therapy initiation. The adjusted ROR of interaction terms of ≥ 70 years × 1 drug was 21.81 (14.56-32.68).

**Conclusion**: Our results suggest that the number of administered anti-infectives and patient age are associated with CDAC. These data may be particularly beneficial to prescribers and would contribute to improving the management of CDAC.

## Introduction

*Clostridium difficile* is a ubiquitous anaerobic bacterium that might cause nosocomial diarrhea. Pathogenic *C. difficile* strains produce two major exotoxins, namely, toxin A and toxin B 1-3. Both toxins stimulate the production of proinflammatory cytokines and exhibit cytotoxic activity. *C. difficile* infection (CDI) presents in a variety of gastrointestinal manifestations, ranging in severity from asymptomatic carrier status to moderate diarrhea and life-threatening pseudomembranous colitis. 1,2,4-7 *C. difficile* is the causative pathogen in 20%-30% of antibiotic-associated diarrhea cases 8-10, in 50%-70% of antibiotic-associated colitis cases, and in more than 90% of antibiotic-associated pseudomembranous colitis cases 11-15. *C. difficile*-associated colitis (CDAC) is responsible for the majority of disease symptoms associated with profuse diarrhea, such as fever, leukocytosis, abdominal pain, and dehydration 5,16.

A recent meta-analysis demonstrated that in Asia, the proportion of CDI in patients with nosocomial diarrhea was 14.8% and the associated mortality was found to be 8.9% 6. Risk factors for CDI include the type of antibiotic, use of a combination of antibiotics, hospitalization, older age, underlying medical conditions, gastrointestinal surgery, and nasogastric tubes 17,18. Antibiotics such as clindamycin, penicillin, fluoroquinolone, and second- and third-generation cephalosporin are typically associated with CDI, but the disease can occur with almost any anti-bacterial agent, including vancomycin and metronidazole, which are commonly used for treatment 15,19,20.

Spontaneous reporting systems (SRSs) such as the Japanese Adverse Drug Event Report (JADER) database have been used in pharmacovigilance assessments. Based on an SRS database, data mining algorithms have been developed to identify drug-associated adverse events (AEs) as signals using reporting odds ratio (ROR) by employing a stepwise method. We previously reported that the concomitant use of drugs might increase the risk of AEs using these approaches 21,22.

Elderly individuals frequently have several chronic health conditions and are administered multiple medications, which is referred to as the number of administered drugs 21,23. The number of administered anti-infectives can lead to drug interactions and may be an important risk factor for CDAC. It has been reported that antibiotic-associated diarrhea incidence was significantly higher in the combined drug-use group than in the monotherapy group 24. To the best of our knowledge, the association between the number of concomitantly used antibiotics and CDAC remains unclear.

The clinical suspicion of CDI is the presentation of diarrhea after the administration of antibiotics shortly after the beginning of treatment and up to 12 weeks after treatment initiation 25-27. Time-to-onset profiles of CDAC derived from the SRS databases have been rarely reported.

The aim of this study was to evaluate the anti-infective-related CDAC profiles by analyzing data from the SRS databases. We assessed the possible relationship among the number of concomitantly used antibiotics, reporting year, sex, and age at CDAC onset using adjusted RORs and a multiple-logistic regression technique. Furthermore, we obtained novel information on the time-to-onset profiles of CDAC relative to the anti-infective therapy initiation.

## Methods

All data from the JADER database were fully anonymized by the regulatory authority of Japan; that is, the Pharmaceuticals and Medical Devices Agency (PMDA). The JADER database is publicly available and can be downloaded from the website of PMDA (www.pmda.go.jp). We assessed the JADER database between April 2004 and June 2018. The JADER database consists of four tables: 1) DEMO (patients' demographic information such as sex, age, and weight); 2) DRUG (drug name, and causality); 3) REAC (AEs and outcome); and, 4) HIST (medical history and primary illness). We integrated a relational database based on four data tables using FileMaker Pro 14 software (FileMaker, Santa Clara, CA, USA). In the DRUG table, the causality of each drug was assigned a code according to its association with AEs, such as “suspected drug,” “concomitant drug,” and “interacting drug.” All cases of “suspected drug,” “concomitant drug,” or “interacting drug” were used in the analyses.

The AE definitions in the JADER database were provided by the Medical Dictionary for Regulatory Activities ver. 19.0/Japanese (MedDRA/J, www.pmrj.jp/jmo/php/indexj.php). We extracted reports of anti-infective-related CDAC using the following preferred terms (PTs): “Clostridium colitis” (PT code: 10058305), “*Clostridium difficile* colitis” (PT code: 10009657), “gastroenteritis clostridial” (PT code: 10017898), and “pseudomembranous colitis” (PT code: 10037128). According to the Anatomical Therapeutic Chemical (ATC) Classification System described by the World Health Organization Collaborating Centre for Drug Statistics Methodology (www.whocc.no/atc_ddd_index/), we verified anti-infectives and subsequently linked to the corresponding ATC classification codes. One hundred and thirty-six anti-infectives were selected and categorized into 32 ATC-drug classes (Table 1).

We calculated the crude RORs by comparing one of the index groups with the reference group. Each ROR was calculated from a two-by-two contingency table; it is the ratio of odds of reporting AEs versus all other events associated with the drug of interest compared with the reporting odds for all other drugs present in the database. The RORs are expressed as point estimates with 95% confidence intervals (CIs). General qualitative judgments were used for signal detection, which depended on signal indices exceeding a predefined threshold. The ROR estimates of < 1 indicate no potential exposure-event associations and estimates of > 1 indicate potential exposure-event safety signals. A signal of the drug-event combination was detected when the lower limit of the 95% CI of the ROR exceeded 1. Furthermore, ≥ 2 cases were required to define the signal 28.

The use of RORs allowed adjustments by multiple-logistic regression analysis and offered the advantage of controlling covariates 29,30. To calculate adjusted RORs, only reports with complete information of reporting year, age, and the number of administered anti-infectives were extracted. To construct the multiple-logistic regression model, reporting year, stratified age groups, and the number of administered anti-infectives were coded. The following multiple-logistic regression model was used in the analysis:

Log (odds) = β_0_ + β_1_Y + β_2_A + β_3_N + β_4_A × N

where, Y is the reporting year, A is the age-stratified group (< 70 years and ≥ 70 years), and N is the number of administered anti-infectives. We evaluated the effects of explanatory variables using a stepwise method 21,31 at a significance level of 0.05 (forward and backward). The contribution of selected variables in the final model was evaluated. The adjusted RORs were calculated using the multiple-logistic regression model. A likelihood ratio test was used to evaluate the effects of explanatory variables.

Most developed world countries and the World Health Organization (WHO) have accepted the chronological age of 65 years as a definition of “elderly” or older person. The description of age was recorded in the data table of DEMO. The reports were stratified by age as follows: ≤19, 20-29, 30-39, 40-49, 50-59, 60-69, 70-79, 80-89, and ≥90 years. For the calculation of the adjusted ROR, the reports were stratified by age as <70 and ≥70 years, because 65 years was categorized into precise 10-year intervals in the JADER database. Neonate, baby, infant, child, young adult, and women in the first-, second-, and third-trimester of pregnancy were categorized into the <70-year-old group. We excluded elderly, adults, and unknown items because these descriptions could not be precisely categorized into the <70 and ≥70-year-old groups. The number of administered anti-infectives was categorized as 1, 2, and ≥3 drugs.

Time-to-onset duration was obtained from the CDAC onset date to the time of the first prescription date for each anti-infective. The median duration, interquartile range, and Weibull shape parameter (WSP) were used to evaluate the time-to-onset profile. The analysis period was 90 days after the first prescription date. The rate of AE occurrence after prescription is assumed to depend on a causal mechanism and often varies with time; in contrast, AEs not associated with the drug should occur at a constant background rate. The WSP test was used for the statistical analysis of time-to-onset data to describe a non-constant ratio of the incidence of AEs. The WSP was used to describe the varying incidence of AEs and to evaluate hazard functions for detecting AEs. The scale parameter α of Weibull distribution determines the scale of the distribution function. A larger scale value (α) stretches data distribution, whereas a smaller scale value shrinks data distribution. The shape parameter β of Weibull distribution determines the shape of the distribution function. The WSP β value indicates the hazard without a reference population; when β is equal to 1, the hazard is estimated to be constant over time. If β is greater than 1 and the 95% CI of β excludes the value of 1, the hazard is considered to increase over time 29,32,33. Information using WSP could be of complementary value for the pharmacovigilance analysis using ROR. All data analyses were performed using JMP 12.0 (SAS Institute, Cary, NC, USA).

## Results

The JADER database contains 534 688 reports submitted between April 2004 and June 2018, and we identified 1222 anti-infective-related CDAC. The top five anti-infectives that cause CDAC-associated AEs were third-generation cephalosporins (ATC code: J01DD, 313 cases), fluoroquinolones (ATC code: J01MA, 201 cases), macrolides (ATC code: J01FA, 146 cases), carbapenems (ATC code: J01DH, 143 cases), and penicillins with extended spectrum (ATC code: J01CA, 103 cases) (Table 2). The drug groups for which the lower limits of 95% CI of RORs were >1 and RORs were >10 were as follows: penicillins with extended spectrum (ATC code: J01CA); combinations of penicillins, including beta-lactamase inhibitors (ATC code: J01CR); first-generation cephalosporins (ATC code: J01DB); second-generation cephalosporins (ATC code: J01DC); third-generation cephalosporins (ATC code: J01DD); fourth-generation cephalosporins (ATC code: J01DE); monobactams (ATC code: J01DF); carbapenems (ATC code: J01DH); other cephalosporins and penems (ATC code: J01DI); macrolides (ATC code: J01FA); lincosamides (ATC code: J01FF); other aminoglycosides (ATC code: J01GB); fluoroquinolones (ATC code: J01MA); and imidazole derivatives (ATC code: J01XD) (Table 2).

**Figure 1:**
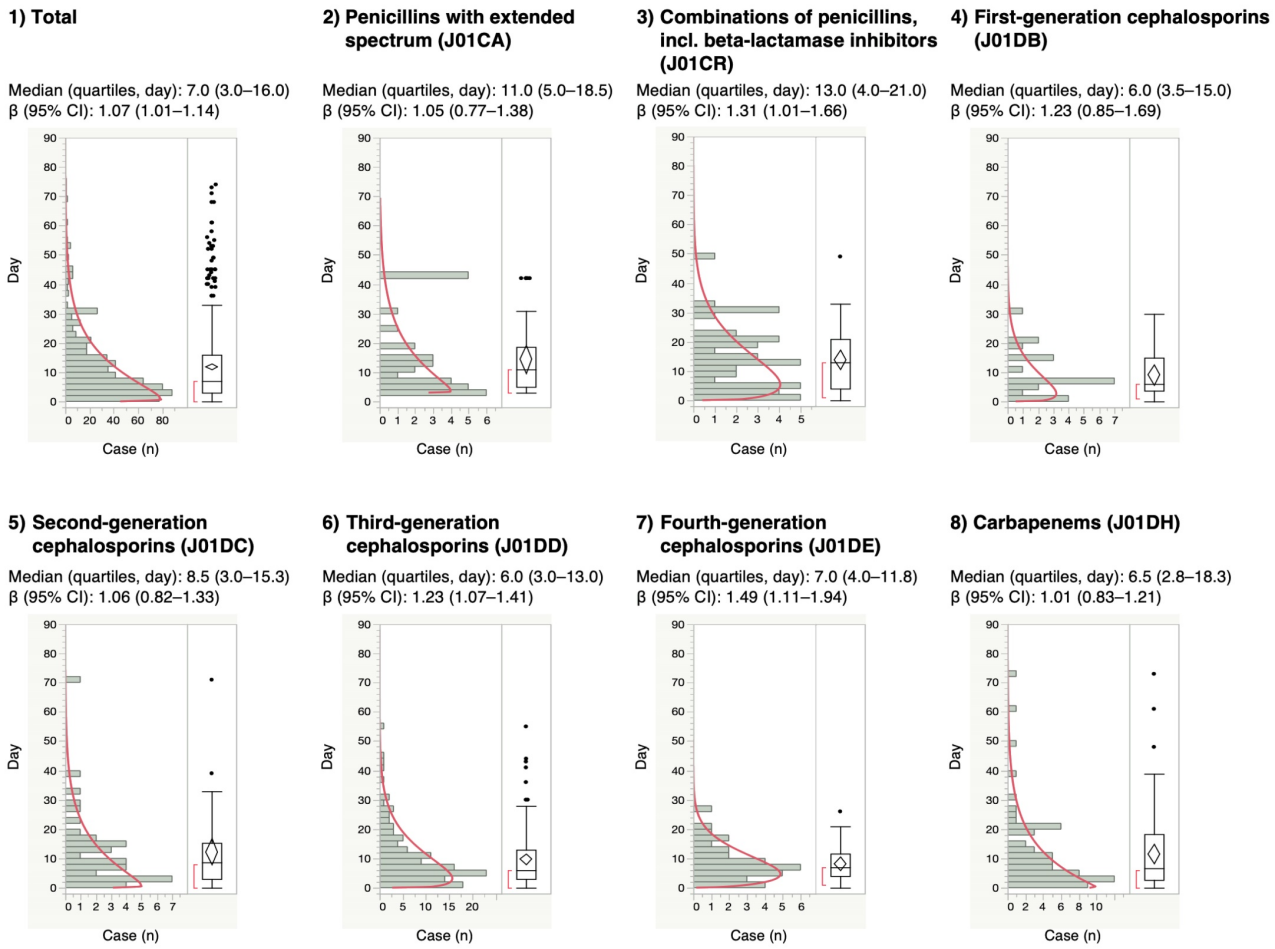

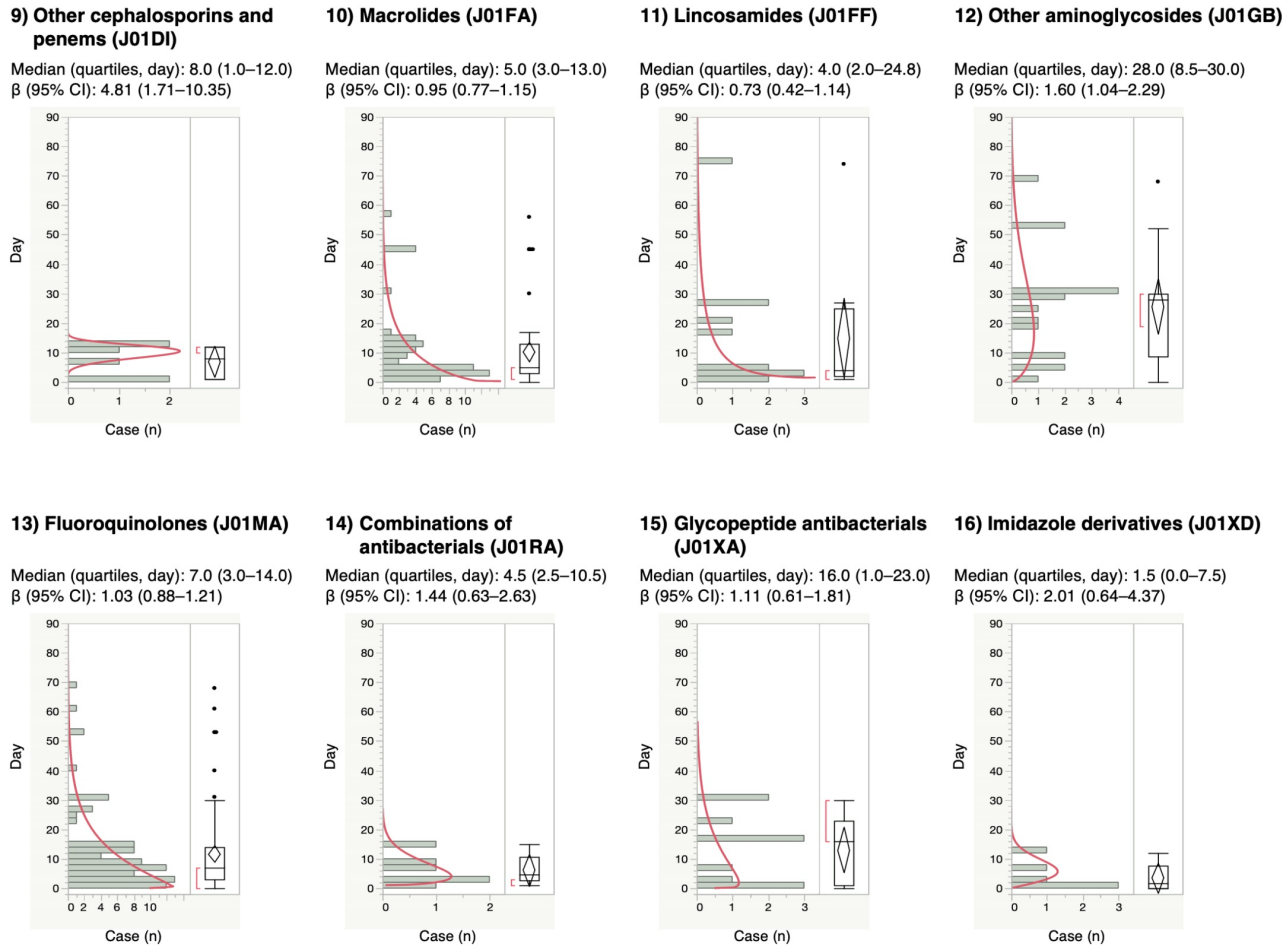

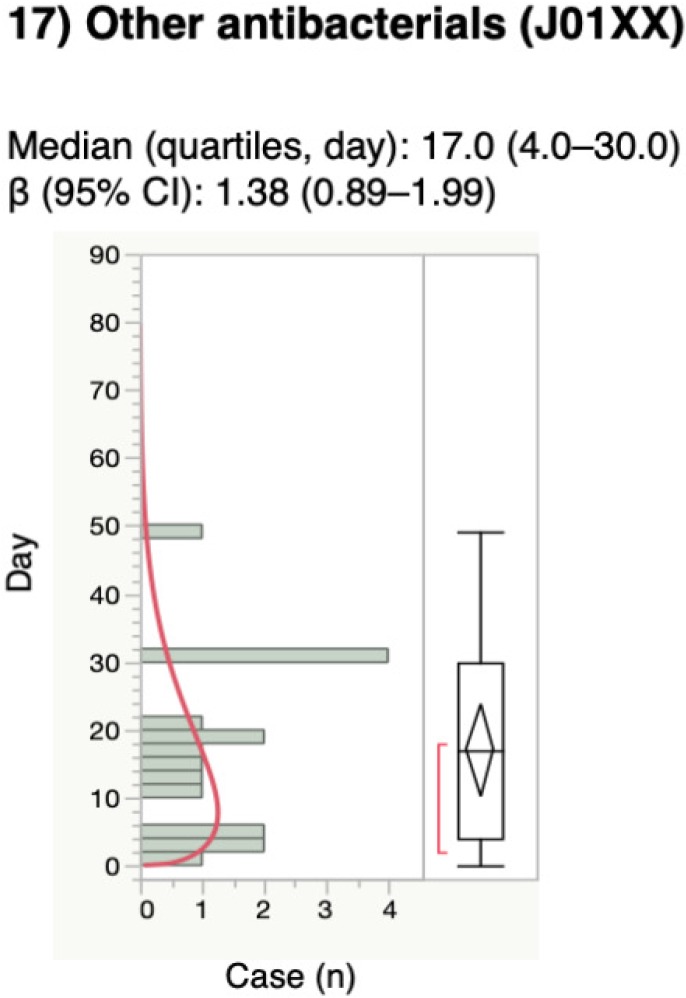
Time-to-onset profiles of anti-infective-related *Clostridium difficile*-associated colitis in the Japanese Adverse Event Report (JADER) database. The records with complete adverse event occurrence and prescription star date were used for the time-to-onset analysis.

For the time-to-onset analysis, we extracted combinations that had complete information on the date of treatment initiation and the date of AE onset. We evaluated 16 anti-infective categories. Figure 1 shows a histogram of the number of CDAC onsets in relation to the number of days after anti-infective treatment initiation (from days 0 to 90). The median period (interquartile range) until CDAC onset caused by anti-infectives was 7.0 (3.0-16.0) days for all anti-infectives (Table 3). The lower limit of the 95% CI of WSP β was >1 for the following drug groups: combinations of penicillins, including beta-lactamase inhibitors (ATC code: J01CR); third-generation cephalosporins (ATC code: J01DD); fourth-generation cephalosporins (ATC code: J01DE); and other cephalosporins, and penems (ATC code: J01DI).

The adjusted RORs and 95% CIs are summarized in Table 4. The results of the model indicated significant contributions of the reporting year (p = 0.0013) and the number of administered anti-infectives (1, 2, and ≥3 drugs, p < 0.0001). The adjusted RORs of the anti-infectives 1, 2, and ≥3 drugs were 8.88 (7.05-11.18), 9.77 (6.89-13.86), and 18.39 (11.85-28.54), respectively. The adjusted ROR of interaction terms of ≥70 years × 1 drug was 21.81 (14.56-32.68).

## Discussion

CDAC, such as pseudomembranous colitis, is a serious colonic disease, which can occur when antibiotics or other agents disrupt the normal colonic flora 34. Almost all classes of anti-infectives increased CDI risk; certain classes including clindamycin, fluoroquinolones, and cephalosporins might have a higher CDI risk than tetracycline 19,34-36. In this study, AE signals indicating an association with CDAC were detected in many categories of anti-infectives such as penicillins with extended spectrum (ATC code: J01CA), first to fourth-generation cephalosporins (ATC code: J01DB, J01DC, J01DD, and J01DE), lincosamides (ATC code: J01FF), and fluoroquinolones (ATC code: J01MA), which agrees with previous findings (Table 2) 36.

CDI can occur after a few days of antibiotic therapy or up to 3 months after use 25-27,37. The median for anti-infective-related CDAC onset was 7 days post-initiation. Furthermore, 47.2% of anti-infective-related CDAC occurred within 7 days of treatment. We did not detect significant differences in time-to-onset profiles among 16 ATC-drug classes of anti-infectives.

The adjusted risk associated with CDI was similar when patients received either a single class of anti-infectives (incidence rate ratio = 2.49, 95% CI 1.59-3.92) or multiple classes of anti-infectives (incidence rate ratio = 2.09, 95% CI 1.23-3.55) 38. Contrarily, in a retrospective cohort study, compared with that for patients who received only 1 antibiotic, the adjusted hazard ratios (HRs) of CDI for those who received 2, 3 or 4, or 5 or more antibiotics were 2.5 (95% CI 1.6-4.0), 3.3 (95% CI 2.2- 5.2), and 9.6 (95% CI 6.1-15.1), respectively 39. Anti-infective-related diarrhea has been reported to be at higher risk in combination therapy than in monotherapy 24. Therefore, we evaluated the risk of CDAC in the real-world clinical setting. The adjusted ROR of anti-infective-related CDAC increased with the number of anti-infectives (Table 4). It is thought that the combination of multiple anti-infectives broadens the antibacterial spectrum and disturbs the intestinal bacterial flora. Avoiding unnecessary antibiotic exposure is often adopted as one of standard preventive strategies for CDI 38.

CDAC is highly common in adults, especially in elderly patients 40. The frequently reported risk factor for recurrent CDI is older age 41-44. We applied the multiple-logistic regression analysis to validate the results. The interaction terms of ≥ 70 years × 1 drug for CDAC was significant in the JADER database.

SRS is a passive reporting system and does not contain detailed patient background to appropriately evaluate an event. SRS is subject to a lack of a comparison group, over-reporting, under-reporting, missing data, and presence of confounders. For example, since the JADER database is based on spontaneous reports, healthcare “professionals” may not usually report CDI to this database. Therefore, the number of cases of CDI might be underestimated.

Proton pump inhibitors and H_2_-blockers have also been identified to increase the risk of acquiring CDI, although the causality and precise mechanism remains unclear. 41,45-48 The effects of these concomitant medications have not been evaluated. The risk of CDI is associated with increasing total dose and days of antibiotic exposure 39,49. It is reported that the incidence rates of CDI following 1-3, 4-6, and 7-11 days of cephalosporin exposure were 1.60, 2.27, and 2.10 times higher than those in no prior receipt, respectively 38. Other risk factors for CDI include the use of a co-administered proton pomp inhibitor and the length of hospital stay 18,24. In this study, we did not evaluate the effect of these factors. Considering the causality restraints of the present analysis, further robust epidemiological studies are recommended.

The association between the number of administered anti-infectives and the onset duration of CDI is interesting. However, when two or more anti-infectives are administered simultaneously, it is difficult to accurately determine the start date of drug administration because the start date cannot be uniquely determined in the JADER database. Therefore, the JADER database is not suitable for analyzing relatively short-term AEs such as CDAC. The median period (interquartile range) until CDAC onset caused by anti-infectives of 1, 2, and ≥3 drugs were apparently 3.0 (1.0-6.0) (n = 191), 5.0 (3.0-8.8) (n = 48), and 10.0 (2.0-21.0) (n = 23) days. We think that this result may not accurately reflect the association between the number of administered anti-infectives and the onset of CDI. Therefore, the interpretation of this result requires great care.

To assist terminology searches, MedDRA is used in post-marketing surveillance. Standardised MedDRA Queries (SMQs) were built by the Maintenance and Support Services Organization. SMQs are groupings of PTs according to the level that relates to a defined medical condition, and the included terms may relate to signs, symptoms, diagnoses, syndromes, physical findings, and laboratory and other physiological test data 50. The grouping of SMQs allows for useful data retrieval and the presentation of relevant individual case safety reports. In the research of CDI in the FDA adverse event reporting system (FAERS), PTs based on pseudomembranous colitis (SMQs) such as “Clostridial infection,” “Clostridial sepsis,” “*Clostridium* bacteraemia,” “*Clostridium* colitis,” “*Clostridium difficile* colitis,” “*Clostridium difficile* infection,” “*Clostridium* test positive,” “Gastroenteritis clostridial,” “Pseudomembranous colitis” and “*Clostridium difficile* sepsis,” which are the lowest level terms, were used to identify CDI cases 36. The choice of term should be made in accordance with the purpose of the study, and thus, our choice of term was focused on CDAC. Therefore, in our study, PTs of “Clostridial infection,” “Clostridial sepsis,” “*Clostridium* bacteremia,” *Clostridium difficile* infection,” “*Clostridium* test positive,” and “*Clostridium difficile* sepsis” were excluded. Generally, with a narrow selection of PTs, the identification of cases is highly likely to represent the condition of interest, and if PTs are selected broadly, the identification of cases might contain all possible cases, including some that may prove to be of little or no interest on closer inspection. The “narrow” scope yields “specificity,” while the “broad” search yields “sensitivity.” 50 We consider that these data suggest the association of certain drugs with CDAC. However, the calculated RORs might vary significantly depending on the selection of PTs, and further validation of these associations is needed.

The diagnosis of CDI is necessary to detect the toxin. However, it is difficult to confirm the diagnosis of CDI directly from the JADER database. We selected PTs for the identification of CDAC based on MedDRA. According to the Introductory Guide MedDRA Version 19.0, each PT is a distinct descriptor (a single medical concept) for a symptom; sign; disease; diagnosis; therapeutic indication; investigation; surgical or medical procedure; or a medical, social, or family history characteristic 51. The JADER database is derived from spontaneous volunteer reporting. The contributors only report AEs according to ICH E2B, the international safety reporting guidelines, and rely on the definitions provided by MedDRA. It might be difficult to confirm the criteria used to define CDAC events by volunteers at the time of reporting. However, the data in the JADER database have been reported by healthcare “professionals.” Therefore, we believe the results are worthy of evaluation.

## Conclusions

To the best of our knowledge, this is the first study to evaluate anti-infective-related CDAC associated with the number of administered anti-infectives and aging using an SRS analysis strategy. Based on the ROR, we demonstrated the potential CDAC risk related to numerous anti-infectives including penicillins with extended spectrum, first- to fourth-generation cephalosporins, lincosamides, and fluoroquinolones. Adjusted RORs demonstrated that the number of administered anti-infectives and aging might be more closely associated with an increasing risk of CDAC. The median period until anti-infective-related CDAC onset was 7 days after therapy initiation. We believe that the data presented in this study will provide guidance for healthcare professionals to improve the care of elderly patients receiving different medications concomitantly.

## Figures and Tables

**Table 1:**
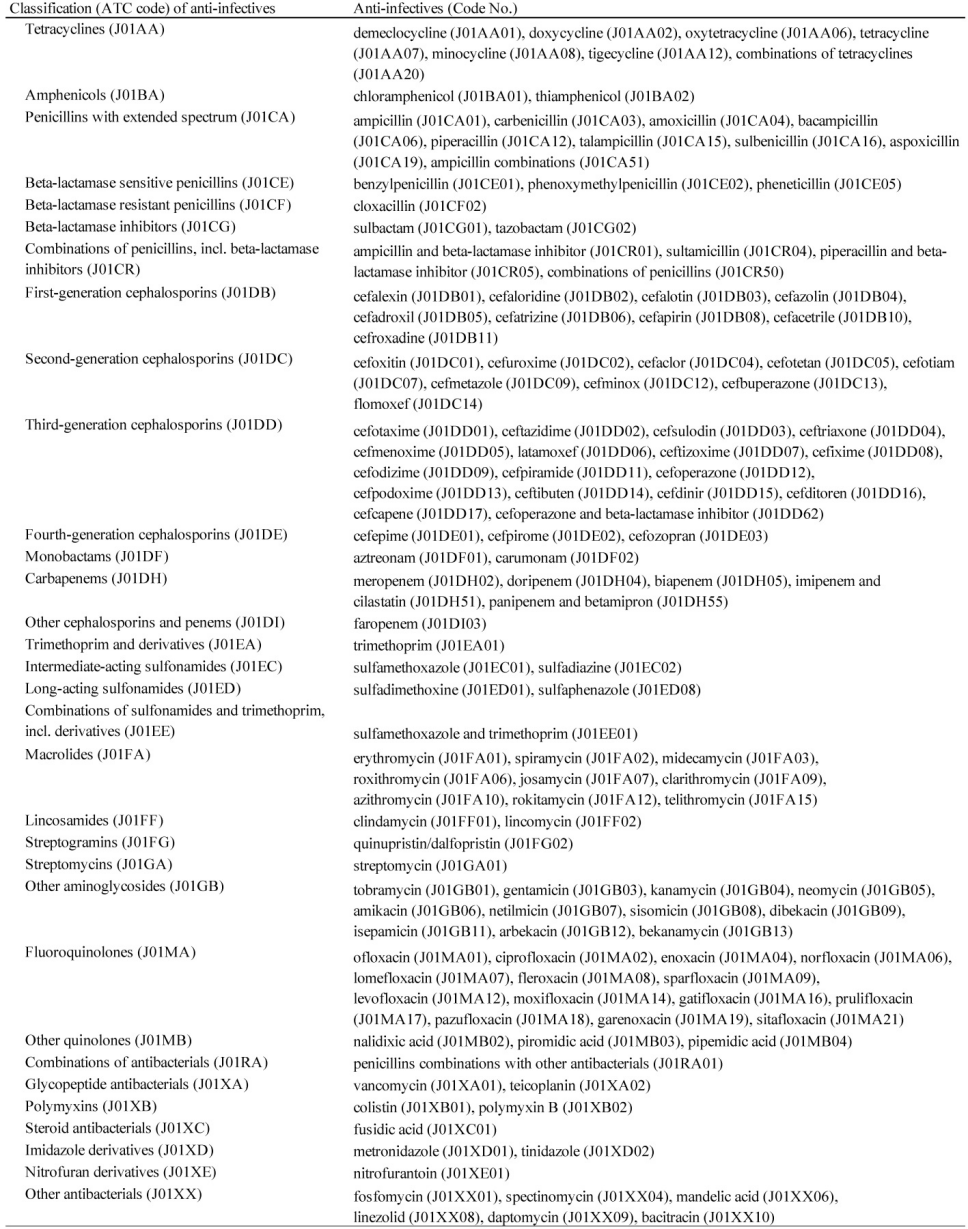
Suspected drugs classified by the Anatomical Therapeutic Chemical classification system and the Defined Daily Dose (ATC/DDD)

**Table 2:**
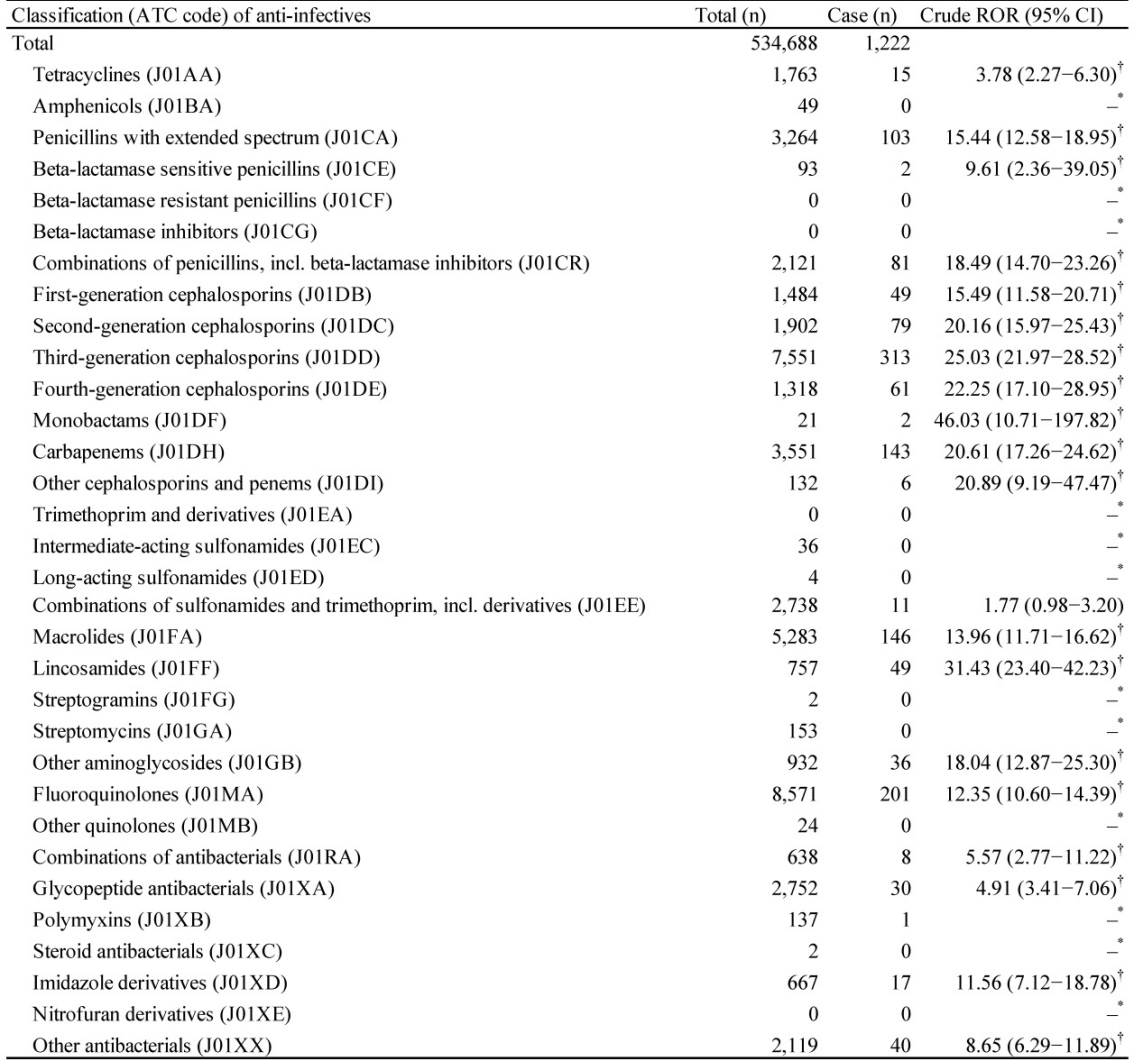
Number of reports and crude reporting odds ratio of *Clostridium difficile*-associated colitis associated with anti-infectives

*Number of cases < 2. †Lower limit of 95% CI > 1.

**Table 3:**
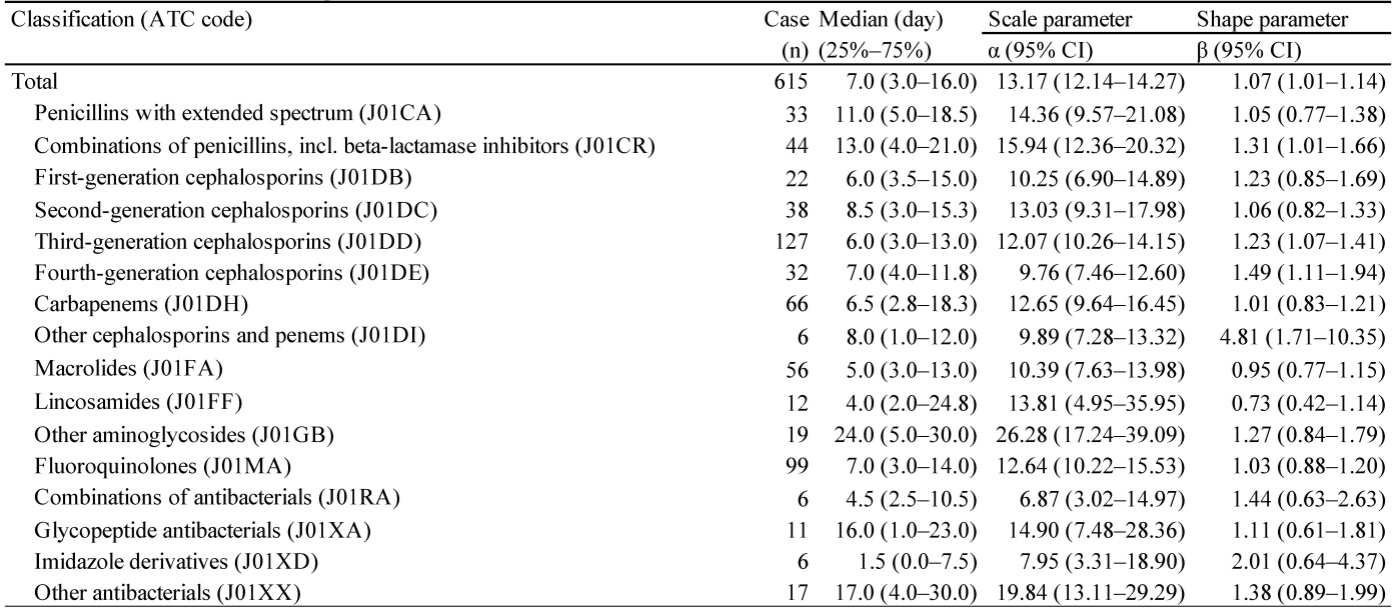
The medians and Weibull parameter of anti-infectives

**Table 4:**
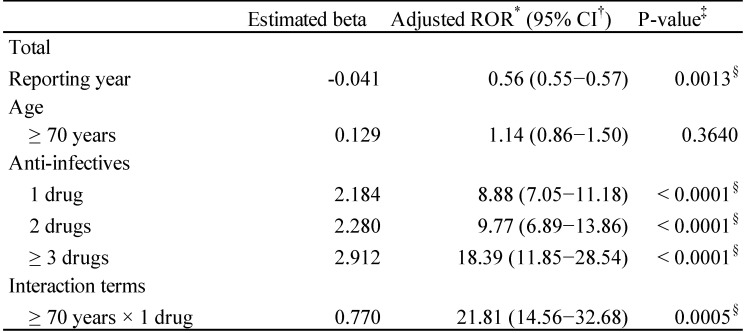
Adjusted reporting odds ratio of *Clostridium difficile*-associated colitis

*Reporting Odds Ratio; †Confidence interval; ‡Probability > Chi-square; δP < 0.05.
